# Supplementary material for: Spatiotemporal transcriptomic analysis during cold ischemic injury to the murine kidney reveals compartment-specific changes
Source: Genome Biol. 2026 Jun 20;27:283. doi: 10.1186/s13059-026-04167-y (PMC13531896; doi:10.1186/s13059-026-04167-y)
Supplement: Supplementary file 1 [file 13059_2026_4167_MOESM1_ESM.pdf]

Spatiotemporal transcriptomic analysis during cold ischemic injury to the murine kidney reveals compartment-specific changes

Supplemental Figures:

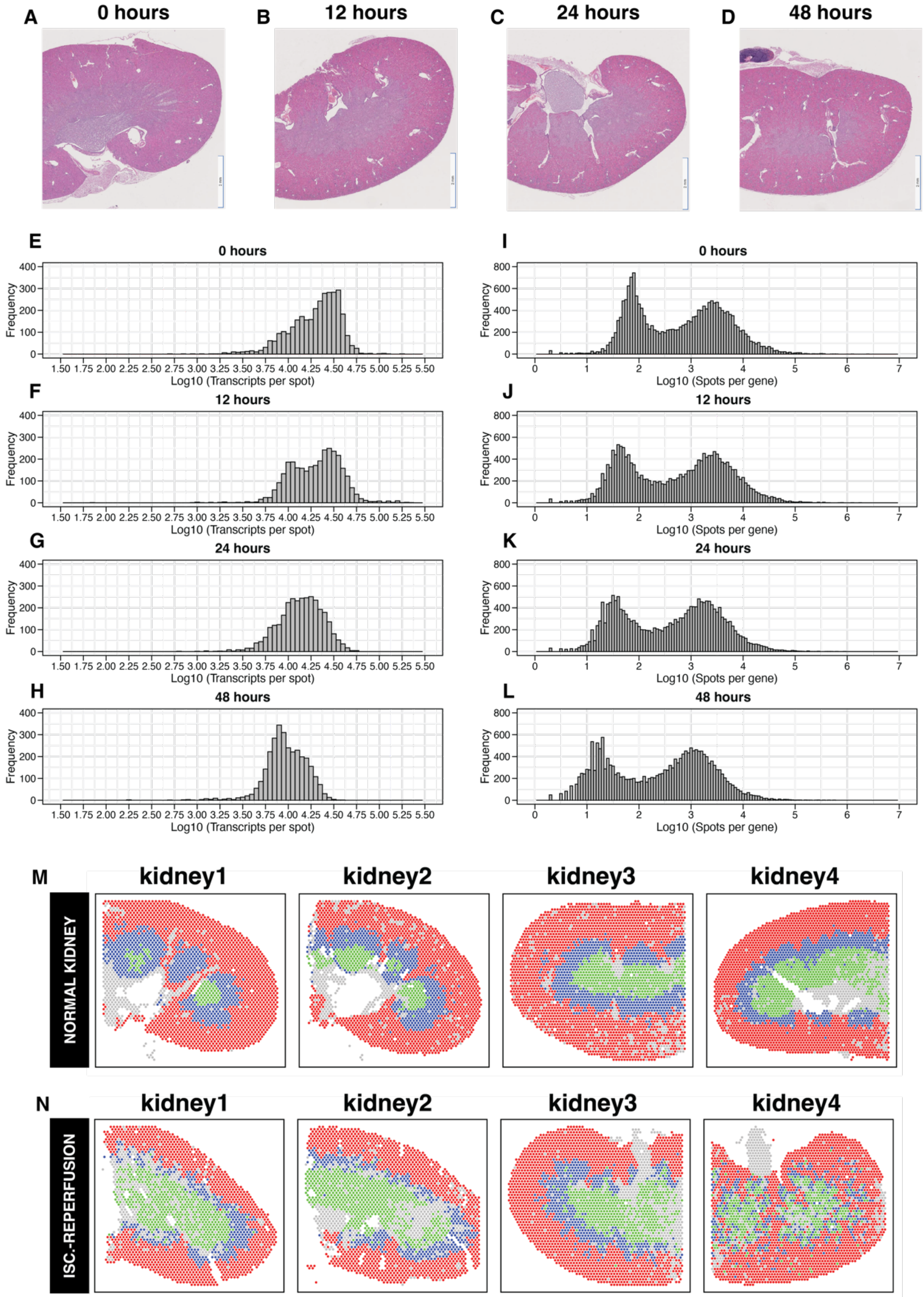

Fig. S1. Tissue specimens and quality control of cold ischemia injury kidneys. H&E-stained images of kidney tissue specimens subjected to **(A)** 0 hours **(B)** 12 hours **(C)** 24 hours and **(D)** 48 hours of cold ischemia injury. Histograms depicting distribution of **(E-H)** transcripts per spot and **(I-L)** spots per gene at time points 0 hours, 12 hours, 24 hours and 48 hours of cold ischemia injury respectively. Annotations highlighting the different compartments namely cortex (red), outer medulla (blue), inner medulla (green) and other (gray), within the normal control (CTRL) kidneys (**M**) and 24-hours warm ischemia reperfusion (AKI24) injury (**N**) tissue specimens.

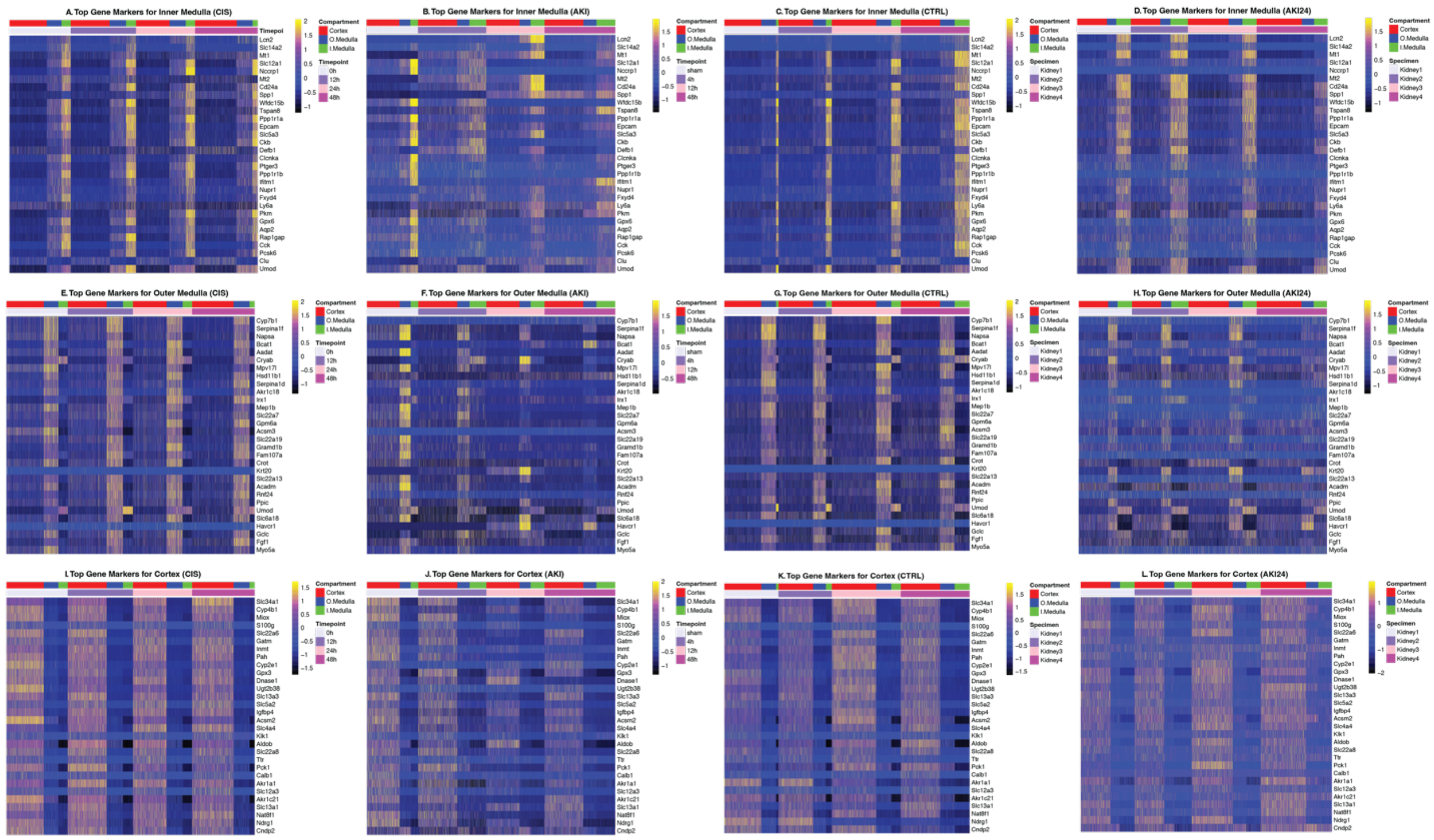

Fig. S2. Heatmap of top compartment-specific marker genes for cold ischemia (CIS), warm ischemia-reperfusion-induced acute kidney injury (AKI), normal control kidneys (CTRL) and 24-hours warm ischemia-reperfusion-induced acute kidney injury (AKI24) injury datasets. Heatmap of top compartment-specific marker genes expression within the different kidney tissue compartments namely **(A,B,C,D)** inner medulla (I.medulla (green)), **(E,F,G,H)** outer medulla (O. medulla (blue)) and **(I,J,K,L)** cortex (red) belonging to different timepoints of cold ischemia (CIS) and warm ischemia-reperfusion (AKI) injury, normal control kidneys (CTRL) and 24-hours warm ischemic-reperfusion injury (AKI24) datasets respectively.

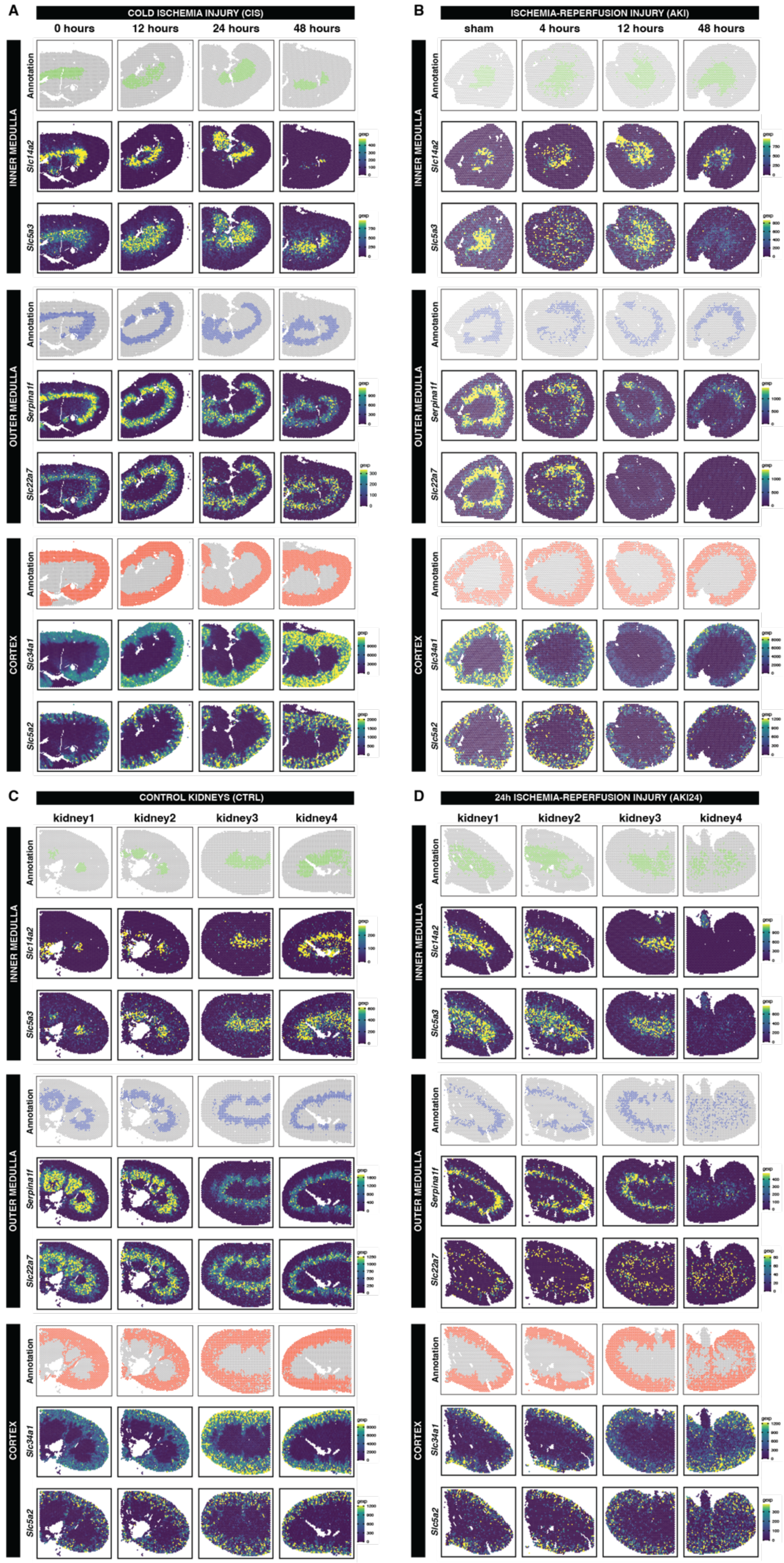

A
COLD ISCHEMIA INJURY (CIS)
0 hours
12 hours
24 hours
48 hours
B
ISCHEMIA-REPERFUSION INJURY (AKI)
sham
4 hours
12 hours
48 hours
C
CONTROL KIDNEYS (CTRL)
kidney1
kidney2
kidney3
kidney4
D
24h ISCHEMIA-REPERFUSION INJURY (AKI24)
kidney1
kidney2
kidney3
kidney4
INNER MEDULLA
OUTER MEDULLA
CORTEX
Annotation
Slc14a2
Slc5a3
Serpina1f
Slc22a7
Slc34a1
Slc5a2

Fig. S3. Spatiotemporal expression patterns of compartment-specific marker genes in different experimental groups. Presentation of inner medulla marker genes (*Slc14a2*, *Slc5a3*); outer medulla marker genes (*Serpina1f, Slc22a7*); cortex marker genes (*Slc34a1, Slc5a2*) within the **(A)** cold ischemia injury (CIS), **(B)** warm ischemia-reperfusion injury (AKI), **(C)** normal control kidneys (CTRL) and **(D)** 24-hours warm ischemia-reperfusion injury (AKI24) kidney tissue specimens. Color coded annotations: red (cortex), blue (outer medulla), green (inner medulla) and other (gray), highlight the location of the different compartments within respective tissue specimens.

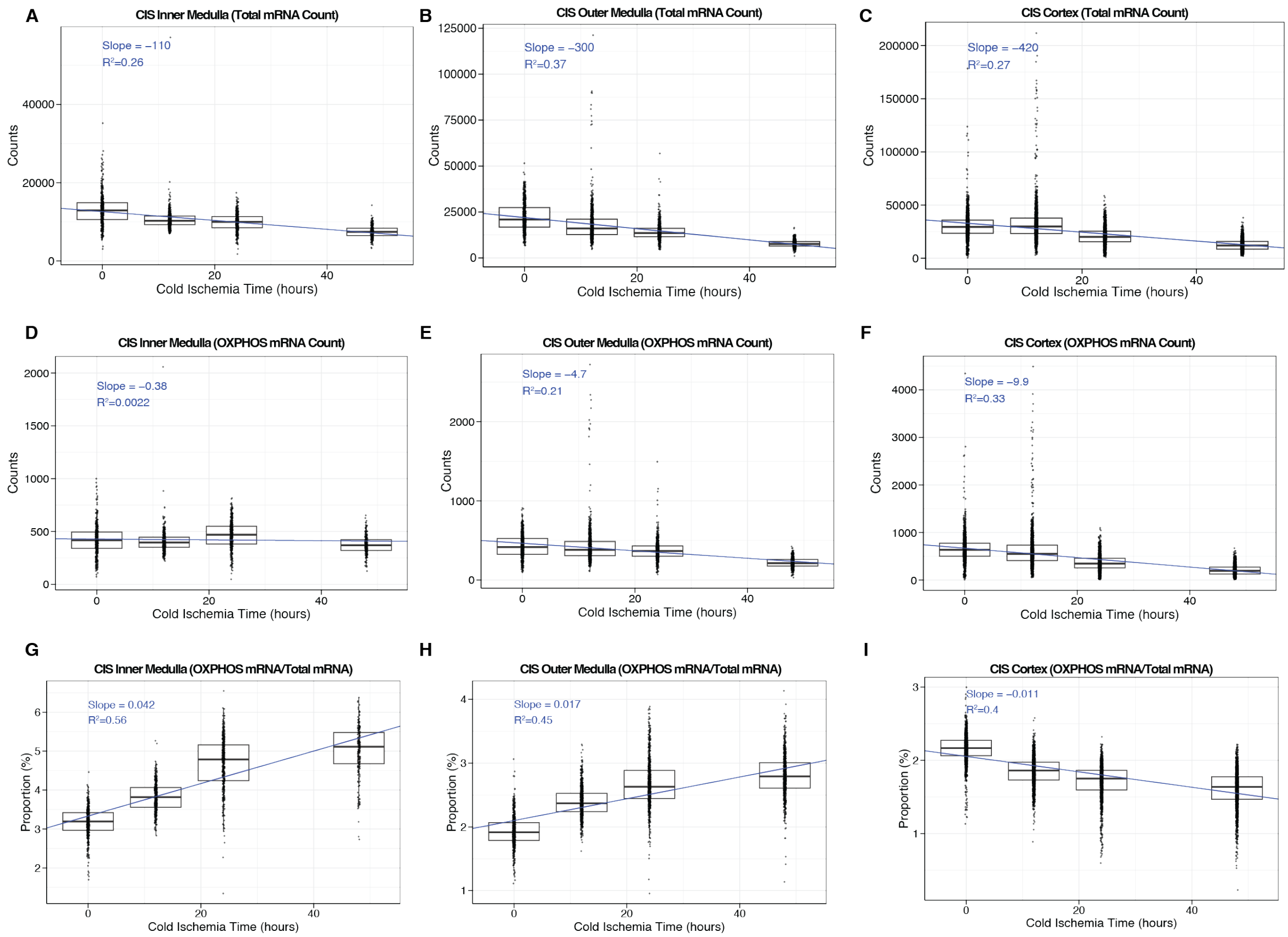

Fig. S4. Compartment-specific proportional enrichment or depletion of OXPHOS genes within cold ischemia kidney tissue. Temporal change in total mRNA count within the **(A)** inner medulla **(B)** outer medulla and **(C)** cortex. Temporal change in OXPHOS related genes within the **(D)** inner medulla **(E)** outer medulla and **(F)** cortex. Temporal change in proportion of OXPHOS related genes with **(G)** inner medulla **(H)** outer medulla and **(I)** cortex. The regression slopes (Slope) and goodness of fit (R^2) of each of the plots is provided at the upper left corner of the respective plots.

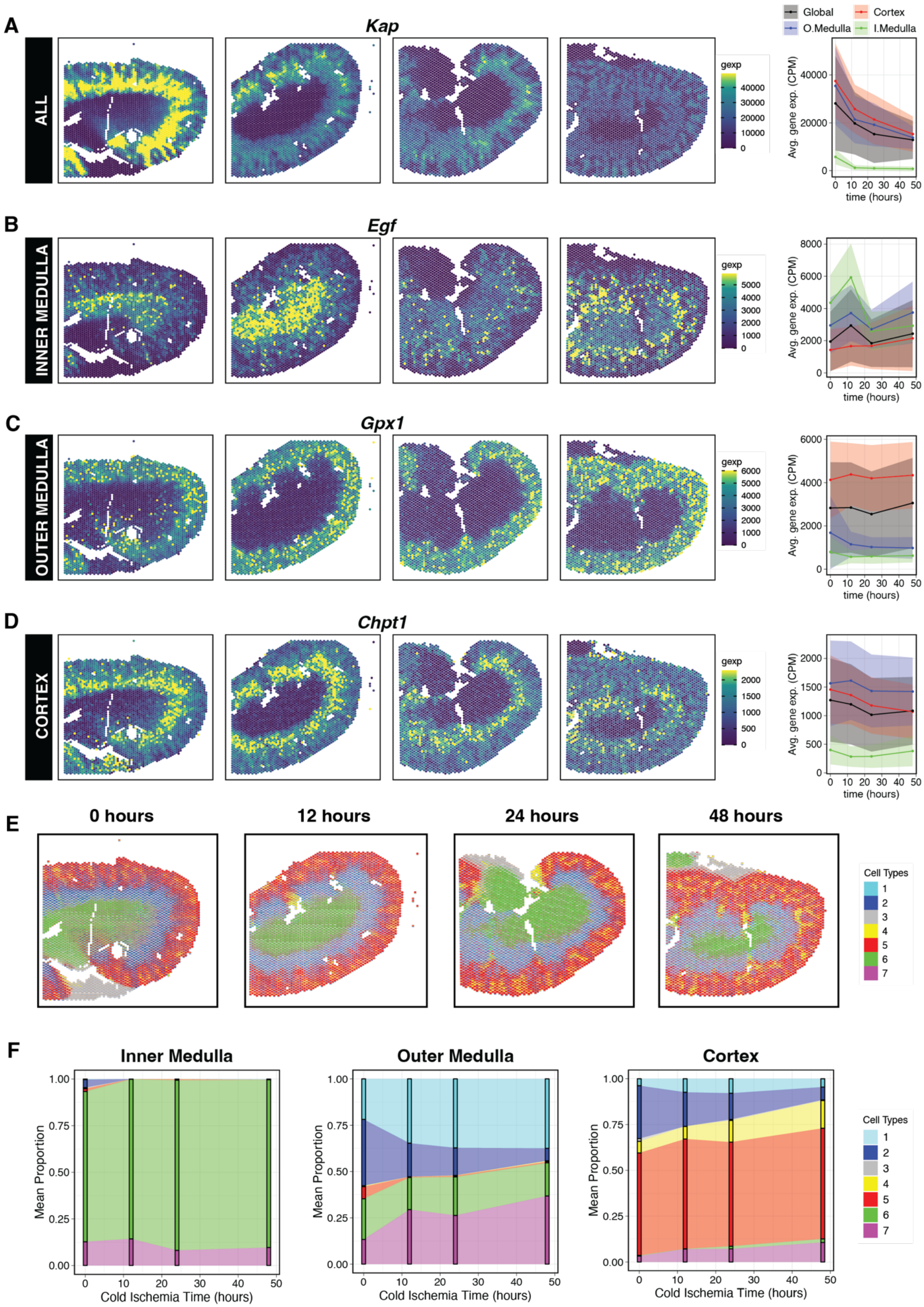

A
Kap
ALL
gexp
Global
Cortex
O.Medulla
I.Medulla
Avg. gene exp. (CPM)
time (hours)
B
Egf
INNER MEDULLA
C
Gpx1
OUTER MEDULLA
D
Chpt1
CORTEX
E
0 hours
12 hours
24 hours
48 hours
Cell Types
F
Inner Medulla
Outer Medulla
Cortex
Mean Proportion
Cold Ischemia Time (hours)

Fig. S5. Representative genes demonstrating compartment-specific downward temporal trend in cold ischemic kidneys and deconvolved cell type changes. While **(A)** *Kap* demonstrated temporally downward trend in all three compartments with varying degrees, **(B)** *Egf,* **(C)** *Gpx1* and **(D)** *Chpt1* were confined to the inner medulla, outer medulla, and cortex respectively. The ribbon plots demonstrate the mean $\pm$ s.d. expression (CPM normalized) of the respective genes within different compartments of the corresponding cold ischemic tissue at different time points. **(E)** Spatial gene expression plots demonstrating spatiotemporal expression of deconvolved cell types (1-7) within cold ischemic kidney tissues. **(F)** Compartment-specific deconvolved cell type composition changes during cold ischemia injury in kidneys.

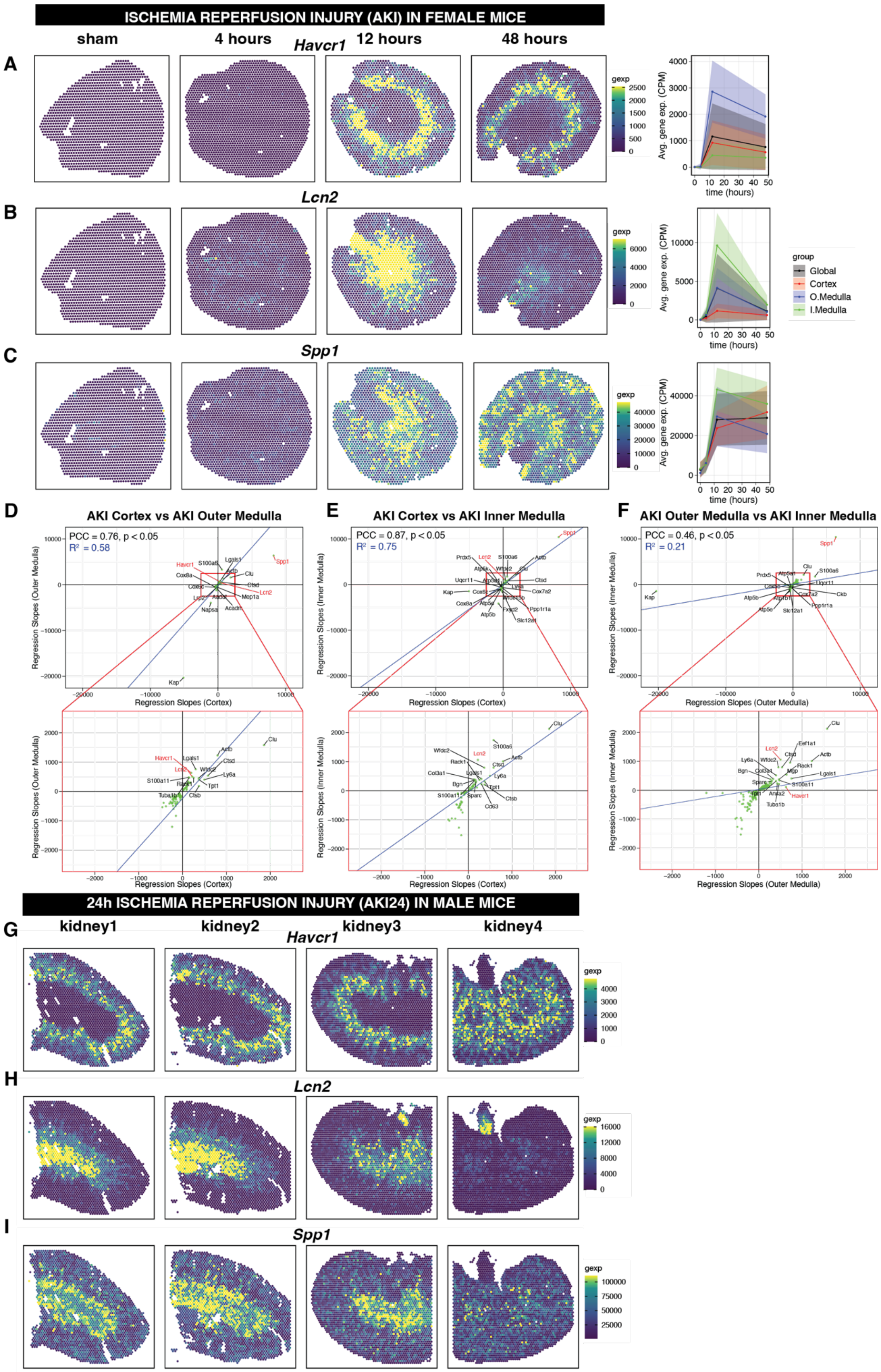
ISCHEMIA REPERFUSION INJURY (AKI) IN FEMALE MICE
sham
4 hours
12 hours
48 hours
A
Havcr1
gexp
2500
2000
1500
1000
500
0
Avg. gene exp. (CPM)
time (hours)
B
Lcn2
6000
4000
2000
group
Global
Cortex
O.Medulla
I.Medulla
C
Spp1
40000
30000
20000
10000
D
AKI Cortex vs AKI Outer Medulla
PCC = 0.76, p < 0.05
R² = 0.58
Regression Slopes (Outer Medulla)
Regression Slopes (Cortex)
E
AKI Cortex vs AKI Inner Medulla
PCC = 0.87, p < 0.05
R² = 0.75
Regression Slopes (Inner Medulla)
F
AKI Outer Medulla vs AKI Inner Medulla
PCC = 0.46, p < 0.05
R² = 0.21
Regression Slopes (Outer Medulla)
24h ISCHEMIA REPERFUSION INJURY (AKI24) IN MALE MICE
kidney1
kidney2
kidney3
kidney4
G
Havcr1
4000
3000
2000
1000
H
Lcn2
16000
12000
8000
4000
I
Spp1
100000
75000
50000
25000

Fig. S6. Spatiotemporal transcriptional dynamics within female and male experimental models of warm ischemia-reperfusion injury (i.e., AKI and AKI24). Spatial plots depicting the spatiotemporal expression of putative AKI marker genes like **(A)** *Havcr1*, **(B)** *Lcn2* and **(C)** *Spp1* within warm ischemia- reperfusion injury (AKI) kidney tissue specimens from female murine models. Scatter plots depicting the correlation analysis of the temporal transcriptional dynamics between the different compartments: **(D)** cortex vs outer medulla **(E)** cortex vs inner medulla and **(F)** outer medulla vs inner medulla of the acute kidney injury (AKI) tissue. Genes exhibiting covarying (green) or divergent (purple) temporal trend within these compartments can be visualized along with their Pearson correlation coefficients (PCC) and linear regression slopes (blue line). AKI marker genes like *Spp1*, *Havcr1* and *Lcn2* within these compartments have been highlighted red. Spatial plots depicting the spatiotemporal expression of **(G)** *Havcr1*, **(H)** *Lcn2* and **(I)** *Spp1* within 24-hours warm ischemia-reperfusion injury (AKI24) kidney tissue specimens from male murine models.

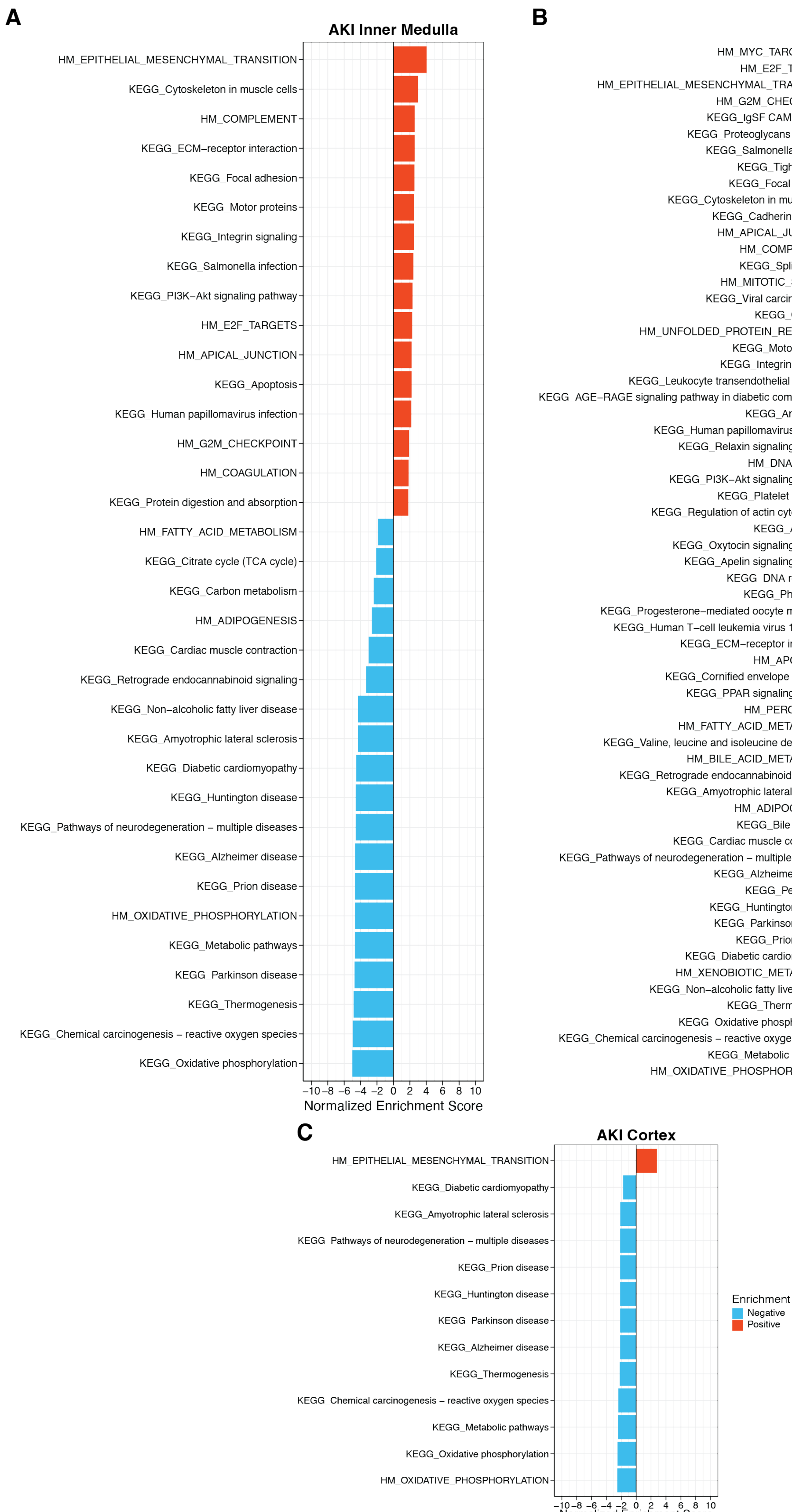

A
AKI Inner Medulla
HM_EPITHELIAL_MESENCHYMAL_TRANSITION
KEGG_Cytoskeleton in muscle cells
HM_COMPLEMENT
KEGG_ECM−receptor interaction
KEGG_Focal adhesion
KEGG_Motor proteins
KEGG_Integrin signaling
KEGG_Salmonella infection
KEGG_PI3K−Akt signaling pathway
HM_E2F_TARGETS
HM_APICAL_JUNCTION
KEGG_Apoptosis
KEGG_Human papillomavirus infection
HM_G2M_CHECKPOINT
HM_COAGULATION
KEGG_Protein digestion and absorption
HM_FATTY_ACID_METABOLISM
KEGG_Citrate cycle (TCA cycle)
KEGG_Carbon metabolism
HM_ADIPOGENESIS
KEGG_Cardiac muscle contraction
KEGG_Retrograde endocannabinoid signaling
KEGG_Non−alcoholic fatty liver disease
KEGG_Amyotrophic lateral sclerosis
KEGG_Diabetic cardiomyopathy
KEGG_Huntington disease
KEGG_Pathways of neurodegeneration − multiple diseases
KEGG_Alzheimer disease
KEGG_Prion disease
HM_OXIDATIVE_PHOSPHORYLATION
KEGG_Metabolic pathways
KEGG_Parkinson disease
KEGG_Thermogenesis
KEGG_Chemical carcinogenesis − reactive oxygen species
KEGG_Oxidative phosphorylation
−10 −8 −6 −4 −2 0 2 4 6 8 10
Normalized Enrichment Score
B
AKI Outer Medulla
HM_MYC_TARGETS_V1
HM_E2F_TARGETS
HM_EPITHELIAL_MESENCHYMAL_TRANSITION
HM_G2M_CHECKPOINT
KEGG_IgSF CAM signaling
KEGG_Proteoglycans in cancer
KEGG_Salmonella infection
KEGG_Tight junction
KEGG_Focal adhesion
KEGG_Cytoskeleton in muscle cells
KEGG_Cadherin signaling
HM_APICAL_JUNCTION
HM_COMPLEMENT
KEGG_Spliceosome
HM_MITOTIC_SPINDLE
KEGG_Viral carcinogenesis
KEGG_Cell cycle
HM_UNFOLDED_PROTEIN_RESPONSE
KEGG_Motor proteins
KEGG_Integrin signaling
KEGG_Leukocyte transendothelial migration
KEGG_AGE−RAGE signaling pathway in diabetic complications
KEGG_Amoebiasis
KEGG_Human papillomavirus infection
KEGG_Relaxin signaling pathway
HM_DNA_REPAIR
KEGG_PI3K−Akt signaling pathway
KEGG_Platelet activation
KEGG_Regulation of actin cytoskeleton
KEGG_Apoptosis
KEGG_Oxytocin signaling pathway
KEGG_Apelin signaling pathway
KEGG_DNA replication
KEGG_Phagosome
KEGG_Progesterone−mediated oocyte maturation
KEGG_Human T−cell leukemia virus 1 infection
KEGG_ECM−receptor interaction
HM_APOPTOSIS
KEGG_Cornified envelope formation
KEGG_PPAR signaling pathway
HM_PEROXISOME
HM_FATTY_ACID_METABOLISM
KEGG_Valine, leucine and isoleucine degradation
HM_BILE_ACID_METABOLISM
KEGG_Retrograde endocannabinoid signaling
KEGG_Amyotrophic lateral sclerosis
HM_ADIPOGENESIS
KEGG_Bile secretion
KEGG_Cardiac muscle contraction
KEGG_Pathways of neurodegeneration − multiple diseases
KEGG_Alzheimer disease
KEGG_Peroxisome
KEGG_Huntington disease
KEGG_Parkinson disease
KEGG_Prion disease
KEGG_Diabetic cardiomyopathy
HM_XENOBIOTIC_METABOLISM
KEGG_Non−alcoholic fatty liver disease
KEGG_Thermogenesis
KEGG_Oxidative phosphorylation
KEGG_Chemical carcinogenesis − reactive oxygen species
KEGG_Metabolic pathways
HM_OXIDATIVE_PHOSPHORYLATION
−10 −8 −6 −4 −2 0 2 4 6 8 10
Normalized Enrichment Score
C
AKI Cortex
HM_EPITHELIAL_MESENCHYMAL_TRANSITION
KEGG_Diabetic cardiomyopathy
KEGG_Amyotrophic lateral sclerosis
KEGG_Pathways of neurodegeneration − multiple diseases
KEGG_Prion disease
KEGG_Huntington disease
KEGG_Parkinson disease
KEGG_Alzheimer disease
KEGG_Thermogenesis
KEGG_Chemical carcinogenesis − reactive oxygen species
KEGG_Metabolic pathways
KEGG_Oxidative phosphorylation
HM_OXIDATIVE_PHOSPHORYLATION
−10 −8 −6 −4 −2 0 2 4 6 8 10
Normalized Enrichment Score
Enrichment
Negative
Positive

Fig. S7. Compartment-specific enrichment of molecular pathways within acute kidney injury (AKI) tissues. Bar plots highlighting all the enriched molecular pathways (Hallmark and KEGG) that demonstrated similar temporal dynamics (enrichment: positive (red), negative (cyan)) within the corresponding **(A)** inner medulla, **(B)** outer medulla and **(C)** cortex of warm ischemia-reperfusion injury (AKI) kidney tissue. Similar, bar plots highlighting all the enriched molecular pathways (KEGG) that demonstrated similar temporal dynamics (enrichment: positive (red), negative (cyan)) within the corresponding **(D)** inner medulla, **(E)** outer medulla and **(F)** cortex of warm ischemia-reperfusion injury (AKI) kidney tissue. (Note: Hallmark pathways have a prefix "HM_" and are in uppercase whereas KEGG pathways have a prefix "KEGG_".)

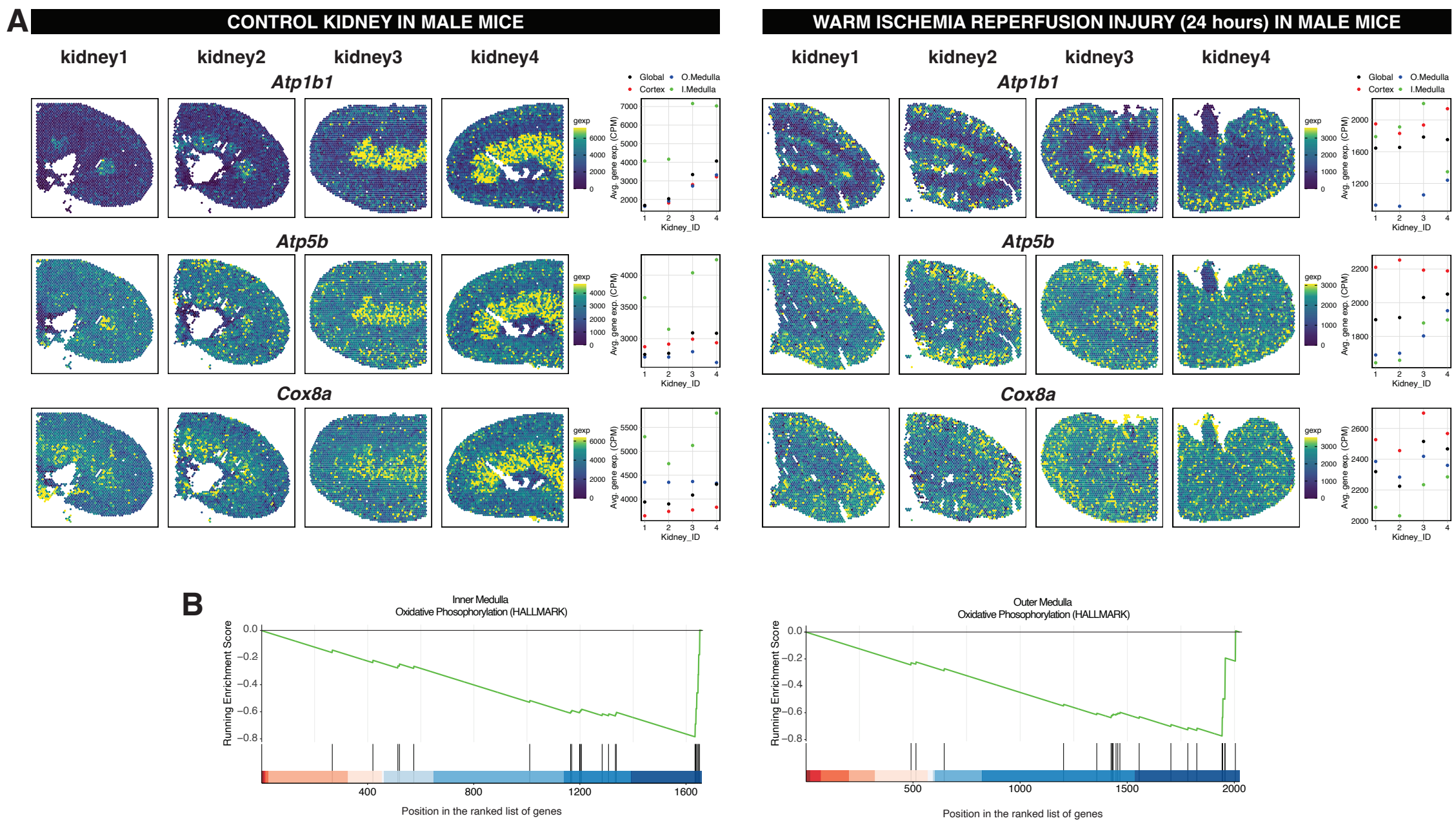

Fig. S8. Differential spatiotemporal expression of OXPHOS genes between the control (CTRL) and warm ischemia injury (24 hours) male kidney. (A) Spatial plots depicting the differential spatiotemporal expression of OXPHOS genes like *Atp1b1, Atp5b* and *Cox8a* between the control and warm ischemia-reperfusion injury (AKI24) kidney tissue specimens from male murine model. **(B)** Running enrichment score plots highlighting differential negative enrichment of OXPHOS pathway (HALLMARK) within the inner medulla of warm ischemia-reperfusion injury (AKI24) male kidney tissue as compared to control male kidney.
