# Supplementary material for: Spatiotemporal transcriptomic analysis during cold ischemic injury to the murine kidney reveals compartment-specific changes
Source: Genome Biol. 2026 Jun 20;27:283. doi: 10.1186/s13059-026-04167-y (PMC13531896; doi:10.1186/s13059-026-04167-y)
Supplement: Supplementary file 2 [file 13059_2026_4167_MOESM2_ESM.pdf]

Supplementary Table Legends

Table S1. Temporally downregulated genes. List of genes that demonstrated a downward temporal trend during cold ischemia injury with the inner medulla, outer medulla, and cortex (indicated by column named "Compartment") of the kidney tissue. Genes are ordered according to their linear regression slopes with the gene demonstrating the maximum temporal change at the top and the minimum at the bottom. R2 represents the goodness of fit (R^2) values obtained from linear regression of the corresponding genes vs cold ischemia time.

Table S2. Temporally upregulated genes. List of genes that demonstrated an upward temporal trend during cold ischemia injury with the inner medulla, outer medulla, and cortex (indicated by column named "Compartment") of the kidney tissue. Genes are ordered according to their linear regression slopes with the gene demonstrating the maximum temporal change at the top and the minimum at the bottom. R2 represents the goodness of fit (R^2) values obtained from linear regression of the corresponding genes vs cold ischemia time.

Table S3. Temporally regulated genes shared across all kidney compartments. List of genes that demonstrated a downward or upward temporal trend (indicated by column named "Trend") during cold ischemia injury shared across all compartments of the kidney tissue.

Table S4. Temporally upregulated genes unique to a particular kidney compartment. List of genes that demonstrated an upward temporal trend during cold ischemia injury that was compartmentally confined to the inner medulla, outer medulla, and cortex (indicated by column named "Compartment") of the kidney tissue. Genes are ordered according to their linear regression slopes with the gene demonstrating the maximum temporal change at the top and the minimum at the bottom.

Table S5. Temporally downregulated genes unique to a particular kidney compartment. List of genes that demonstrated a downward temporal trend during cold ischemia injury that was compartmentally confined to the inner medulla, outer medulla, and cortex (indicated by column named "Compartment") of the kidney tissue. Genes are ordered according to their linear regression slopes with the gene demonstrating the maximum temporal change at the top and the minimum at the bottom.

Table S6. Covarying transcriptional trends of genes between different compartments of kidney during cold ischemia (CIS) injury. List of genes that demonstrated covarying transcriptional trend within the different compartments: cortex vs outer medulla, cortex vs inner medulla and outer medulla vs inner medulla (indicated by column named "Comparison") of the CIS kidney tissue. Each sheet contains the linear regression slope value of the genes within the compartments being compared. A positive slope value indicates upregulation while a negative value indicates a downregulation.

Table S7. Divergent transcriptional trends of genes between different compartments of kidney during cold ischemia (CIS) injury. List of genes that demonstrated divergent transcriptional trend within the different compartments: cortex vs outer medulla, cortex vs inner medulla and outer medulla vs inner medulla (indicated by column named "Compariosn") of the CIS kidney tissue. Each sheet contains the linear regression slope value of the genes within the compartments being compared. A positive slope value indicates upregulation while a negative value indicates a downregulation.

Table S8. Ranked list of genes associated with major deconvoluted cell types within kidney tissue during cold ischemia (CIS) injury. List of top 30 genes associated with the 7 major deconvoluted cell types (indicated by column named "CellType") within kidney tissue during cold ischemia injury.

Table S9. Enriched molecular (GO) pathways within different compartments of kidney during cold ischemia (CIS) injury. List of enriched molecular pathways (GO) associated with top genes for 7 major deconvolved cell types (indicated by column named "CellType") within the kidney tissue during cold ischemia injury. Molecular pathways are ordered according to their normalized enrichment scores (NES) obtained from gene set enrichment analysis with pathways with highest NES at the top and minimum NES at the bottom. Corresponding adjusted p-values (p.adjust) and genes with significant contribution to the NES (Leading Edge Genes) for the respective enriched pathways are also provided. Pathways with p.adjust<0.05 are not included in the list. Enriched molecular pathways list belonging to each deconvoluted cell types are provided.

Table S10. Enriched molecular (KEGG) pathways within different compartments of kidney during cold ischemia (CIS) injury. List of enriched molecular pathways (KEGG) associated with genes that demonstrated a significant temporal trend (either upward or downward) during cold ischemia injury within the inner medulla, outer medulla, and cortex (indicated by column named "Compartment") of the CIS kidney tissue. Molecular pathways are ordered according to their normalized enrichment scores (NES) obtained from gene set enrichment analysis with pathways with highest NES at the top and minimum NES at the bottom. Corresponding adjusted p-values (p.adjust) and genes with significant contribution to the NES (Leading Edge Genes) for the respective enriched pathways are also provided. Pathways with p.adjust<0.05 are not included in the list.

Table S11. Enriched molecular (Hallmark gene sets of MSigDB) pathways within different compartments of kidney during cold ischemia (CIS) injury. List of enriched molecular pathways (Hallmark gene sets of MSigDB) associated with genes that demonstrated a significant temporal trend (either upward or downward) during cold ischemia injury within the inner medulla, outer medulla, and cortex (indicated by column named "Compartment") of the CIS kidney tissue. Molecular pathways are ordered according to their normalized enrichment scores (NES) obtained from gene set enrichment analysis with pathways with highest NES at the top and minimum NES at the bottom. Corresponding

adjusted p-values (p.adjust) and genes with significant contribution to the NES (Leading Edge Genes) for the respective enriched pathways are also provided. Pathways with p.adjust<0.05 are not included in the list.

Table S12. Enriched molecular (KEGG) pathways within different compartments of kidney during warm ischemia-reperfusion (AKI) injury. List of enriched molecular pathways (KEGG) associated with genes that demonstrated a significant temporal trend (either upward or downward) during warm ischemia-reperfusion injury within the inner medulla, outer medulla and cortex (indicated by column named "Compartment") of the AKI kidney tissue. Molecular pathways are ordered according to their normalized enrichment scores (NES) obtained from gene set enrichment analysis with pathways with highest NES at the top and minimum NES at the bottom. Corresponding adjusted p-values (p.adjust) and genes with significant contribution to the NES (Leading Edge Genes) for the respective enriched pathways are also provided. Pathways with p.adjust<0.05 are not included in the list.

Table S13. Enriched molecular (Hallmark gene sets of MSigDB) pathways within different compartments of kidney during warm ischemia-reperfusion (AKI) injury. List of enriched molecular pathways (Hallmark gene sets of MSigDB) associated with genes that demonstrated a significant temporal trend (either upward or downward) during warm ischemia-reperfusion injury within the inner medulla, outer medulla, and cortex (indicated by column named "Compartment") of the AKI kidney tissue. Molecular pathways are ordered according to their normalized enrichment scores (NES) obtained from gene set enrichment analysis with pathways with highest NES at the top and minimum NES at the bottom. Corresponding adjusted p-values (p.adjust) and genes with significant contribution to the NES (Leading Edge Genes) for the respective enriched pathways are also provided. Pathways with p.adjust<0.05 are not included in the list.

Table S14. Covarying molecular (KEGG and Hallmark) pathways within corresponding compartments of CIS and AKI kidney tissue. List of enriched molecular pathways (KEGG and HALLMARK) within the CIS and AKI tissue that demonstrated covarying trend within the corresponding inner medulla, outer medulla, and cortex (indicated by column named "Compartment") of the CIS and AKI tissue. Each sheet contains the normalized enrichment score (NES) of the covarying genes within the respective CIS and AKI tissue. Pathways with "HALLMARK_" as prefix belong to Hallmark geneset.

Table S15. Divergent molecular (KEGG and Hallmark) pathways within corresponding compartments of CIS and AKI kidney tissue. List of enriched molecular pathways (KEGG and HALLMARK) within the CIS and AKI tissue that demonstrated divergent trend within the corresponding inner medulla, outer medulla, and cortex (indicated by column named "Compartment") of the CIS and AKI tissue. Each sheet contains the normalized enrichment score (NES) of the divergent genes within the respective CIS and AKI tissue. Pathways with "HALLMARK_" as prefix belong to Hallmark geneset.
